# Obesity, White Adipose Tissue, and Adipokines Signaling in Male Reproduction

**DOI:** 10.1002/mnfr.70054

**Published:** 2025-04-08

**Authors:** Fabiane Ferreira Martins, Maria do Socorro Medeiros Amarante, Daiana Santana Oliveira, Isabela Macedo Lopes Vasques‐Monteiro, Vanessa Souza‐Mello, Julio Beltrame Daleprane, Christina da Silva Camillo

**Affiliations:** ^1^ Department of Morphology Federal University of Rio Grande do Norte Natal Rio Grande do Norte Brazil; ^2^ Laboratory of Morphometry Metabolism and Cardiovascular Diseases Biomedical Center Institute of Biology Rio de Janeiro State University Rio de Janeiro Brazil; ^3^ Department of Basic and Experimental Nutrition Laboratory for Studies of Interactions Between Nutrition and Genetics LEING Rio de Janeiro State University Rio de Janeiro Brazil

**Keywords:** adipokines, adipose tissue, male reproduction, obesity, testis

## Abstract

Currently, obesity is a global pandemic characterized by systemic metabolic complications that negatively impact several organs, including white adipose tissue (WAT) and the tissues of the male reproductive system. Since the discovery of leptin in 1994, WAT has been recognized as a dynamic endocrine organ for secreting a series of molecules with hormonal functions, collectively called adipokines. The link between obesity, WAT, adipokines, and the male reproductive system is direct and little explored. With changes in nutritional status, WAT undergoes morphofunctional changes, and the secretion of adipokines is altered, negatively impacting reproductive mechanisms, including steroidogenesis and spermatogenesis. In this review, we address in an updated way the structural and functional characteristics of WAT as well as the link between obesity and changes in the signaling pathways of the adipokines leptin, adiponectin, resistin, visfatin, apelin, chemerin, omentin‐1, vaspin, and asprosin in male reproduction. Understanding the relationship between obesity, these adipokines, and reproductive dysfunction can contribute to new strategies for the treatment of subfertility and male infertility.

## Graphical abstract

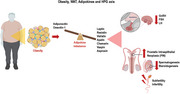

**Abbreviations**
3β‐HSD: 3β‐hydroxysteroid dehydrogenase
AMPK: AMP‐activated protein kinase
APLN: proapelin gene
ARs: androgen receptors
ATGL: adipose triglyceride lipase
BMI: body mass index
CAP1: adenylyl cyclase‐associated protein 1
CCRL2: C‐C motif chemokine receptor‐like 2
cFos: AP‐1, transcription factor subunit
cJUN: c‐Jun amino terminal kinases
CMKLR1: chemokine‐like receptor 1
Dax1: dosage‐sensitive sex reversal, adrenal hypoplasia critical region, on chromosome X, gene 1
DM2: type 2 diabetes mellitus
ERs: estrogen receptors
FSH: follicle‐stimulating hormone
GnRH: gonadotropin‐releasing hormone
GPR1: G‐protein‐coupled receptor 1
HPG: hypothalamic‐pituitary‐gonadal axis
HSL: hormone‐sensitive lipase
IL: interleukin
JAK2: Janus kinase 2
LH: luteinizing hormone
LPL: lipoprotein lipase
MAPK: mitogen‐activated protein kinase
MAPK ERK1/2: mitogen‐activated protein kinase/extracellular signal‐regulated kinase 1/2
MCP‐1: monocyte chemoattractant protein1
NAMPT: nicotinamide phosphoribosyltransferase
NFκB: nuclear factor kappaB
Nur77: orphan nuclear receptor
OB‐Rs: leptin receptors
PIN: prostatic intraepithelial neoplasia
PPAR‐α: peroxisome proliferator‐activated receptor‐α
SOCS3: suppressor of cytokine signaling 3
STAR: steroidogenic acute regulatory protein
STAT3: signal transducer and activator of transcription 3
sWAT: subcutaneous white adipose tissue
TGs: triacylglycerols
TLR4: toll‐like receptor 4
TNF‐α: tumor necrosis factor alpha
TSPO: translocator protein
VLDL: low‐density lipoproteins
vWAT: visceral white adipose tissue
WAT: white adipose tissue
WHO: World Health Organization

## 1 Introduction

Obesity is currently considered a global pandemic that is emerging as a socioeconomic and public health challenge [1]. According to the World Health Organization (WHO), the global prevalence of obesity has doubled since the 1980s. In 2022, 2.5 million adults were overweight, and 890 million were living with obesity [2]. The WHO defines the criteria for obesity as individuals who have excessive accumulation of fat that can harm health and who have a body mass index (BMI) ≥ 30 kg/m^2^ [3]. Obesity and its metabolic complications substantially impair the functioning of several target tissues, including the heart, pancreas, liver, different adipose tissue depots, and reproductive organs [4].

Several studies show a close association between obesity and male reproductive dysfunction. Obesity represents one of the leading causes of secondary hypogonadism in men. This condition is characterized by failures in the regulation of the hypothalamic‐pituitary‐gonadal (HPG) axis and the reduction in testosterone levels accompanied by defects in spermatogenesis, changes in semen quality, erectile dysfunction, and loss of libido [5, 6].

In this context, white adipose tissue (WAT) has received significant attention. Until the discovery of leptin in 1994, WAT assumed the position of an energy reserve and thermal insulation tissue. Since then, WAT has been recognized as a dynamic tissue with a high endocrine activity that secretes a series of hormones collectively called adipokines [7, 8]. Adipokines are involved in numerous systemic physiological processes, including regulating male reproductive health [9]. Under the pathogenesis of obesity, WAT and the secretion of adipokines are affected, resulting in direct consequences for reproductive functions [10].

In this review, we address in an updated way the interaction between WAT, adipokines, and male reproductive organs in the physiological and pathological state (affected by obesity). Understanding the functioning of reproductive functions in a healthy state and obesity is fundamental for establishing new therapeutic routes.

## 2 Obesity, WAT, and Adipokines in Male Reproduction

**FIGURE 1:**
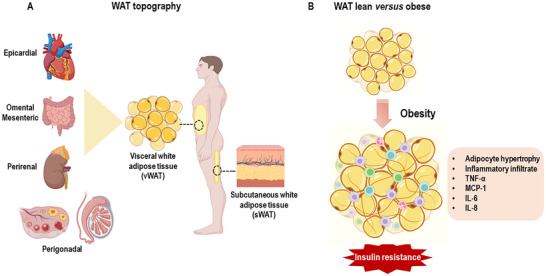
Location of the different depots of white adipose tissue (WAT) in the human body. (A) Structural and functional changes of WAT under obesity. (B) The adipose tissue of lean individuals has small, insulin‐sensitive adipocytes. In contrast, the adipose tissue of obese individuals presents hypertrophied, insulin‐resistant adipocytes with an infiltrate of inflammatory cells and secretion of pro‐inflammatory cytokines (Figure created with website https://app.biorender.com). IL‐6, interleukin‐6; IL‐8, interleukin‐8; MCP‐1, monocyte chemoattractant protein1; TNF‐α, tumor necrosis factor‐alpha.

WAT is widely distributed in the body and is organized in a cellular arrangement where one‐third is composed of unilocular adipocytes and the remainder is represented by preadipocytes, fibroblasts, cells of the immune system (macrophages, eosinophils, neutrophils, and mast cells), blood vessels, nerves, and stromal elements (collagen and elastic fibers) [11]. Topographically, WAT is found under the skin, constituting the subcutaneous depot (sWAT) and in specific compartments called visceral depot (vWAT) that are deposited around the heart and intra‐abdominal organs (omental, mesenteric, retroperitoneal, perirenal, and perigonadal) [12] (Figure 1A).

The primary function of WAT is the regulation of lipid metabolism through the processes of lipogenesis and lipolysis. In lipogenesis, under dietary conditions of excessive energy intake and/or reduced energy expenditure, WAT stores excess fuel as neutral triacylglycerols (TGs). In this process, lipoprotein lipase (LPL) present in the endothelium of blood vessels hydrolyzes the TGs of chylomicrons and low‐density lipoproteins (VLDL), releasing fatty acids that are transported into the adipocytes, which are aggregated with glycerol molecules, forming stable droplets of TGs. Conversely, in situations of nutrient scarcity or greater energy demand, lipolysis occurs; TGs are hydrolyzed by adipose triglyceride lipase (ATGL) and hormone‐sensitive lipase (HSL), where fatty acids are transported through the bloodstream and directed to tissues as energy substrates for mitochondrial beta‐oxidation [13, 14].

Recently, WAT has been highlighted beyond its classical role as an energy storage organ to a biologically dynamic and functional endocrine organ that secretes various signaling molecules, the adipokines. These molecules act in an autocrine, paracrine, and endocrine manner, modulating multiple cellular mechanisms [15]. In physiological aspects, adipokines place WAT in the position of central coordinator of metabolism, regulating energy intake and expenditure through action on the orexigenic and anorexigenic centers of the hypothalamus and modulating body metabolic homeostasis by acting directly on target organs such as the heart, liver, pancreas, skeletal muscle, and male reproductive organs [16, 17].

Obesity and excess lipids converge to an increase in lipogenesis and a reduction in energy expenditure, in addition to generating structural and phenotypic changes in WAT characterized by dysfunctional and inflamed hypertrophic adipocytes. Overnutrition triggers insulin resistance and uncontrolled inflammatory responses in WAT, leading to chronic low‐grade inflammation with increased inflammatory cell infiltration and secretion of pro‐inflammatory cytokines (tumor necrosis factor alpha, TNF‐α; monocyte chemoattractant protein1, MCP‐1; interleukin‐6, IL‐6; and interleukin‐8, IL‐8) [18, 19] (Figure 1B). This morphofunctional distortion of WAT leads to changes in the secretion profile of adipokines and their signaling routes [20]. Currently, more than 20 types of adipokines that exert systemic actions have been identified and recognized [21]. In the context of reproduction and male reproductive health, leptin, adiponectin, resistin, visfatin, apelin, chemerin, omentin‐1, vaspin, and asprosin are adipokines that are intimately involved with the regulation of spermatogenesis and steroidogenesis, and in the state of weight gain and obesity, they deserve great attention.

## 3 Leptin

**FIGURE 2:**
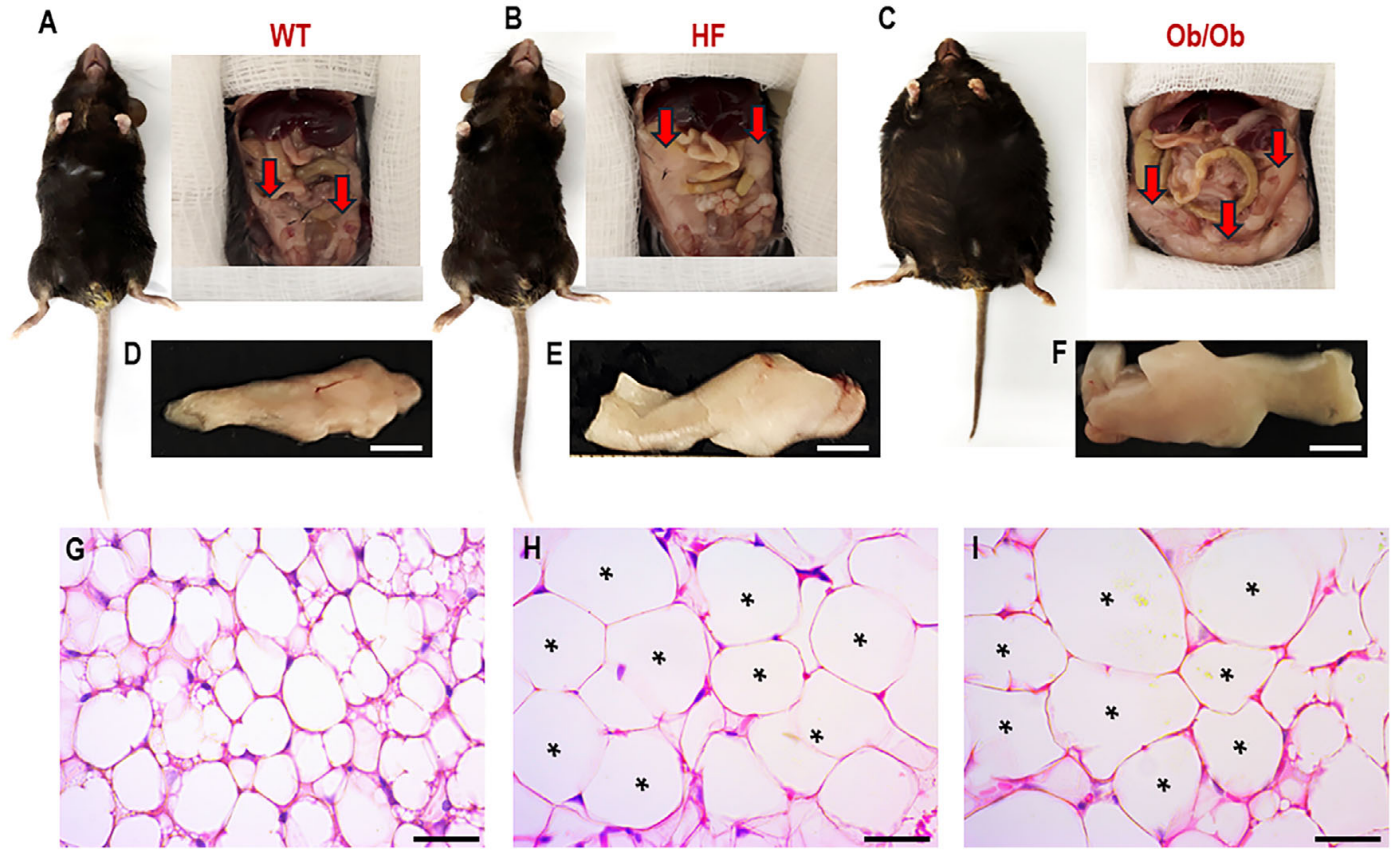
Body and adiposity differences in different models of obesity. Lean mice (WT) (A, D, G), obese mice via induced diet (HF) (B, E, H), obese leptin knockout mice (Ob/Ob) (C, F, I). Compared to WT mice, HF mice develop resistance to leptin and defects in its signaling, showing more significant deposition of gonadal adipose tissue (arrows, B and E) and hypertrophied adipocytes (asterisks, H). Ob/Ob mice do not express leptin; as a result, they present exacerbated adiposity (arrows, C and F) and adipocytes with a greater degree of hypertrophy compared to HF mice (asterisks, I). Hematoxylin‐eosin stain.

**FIGURE 3:**
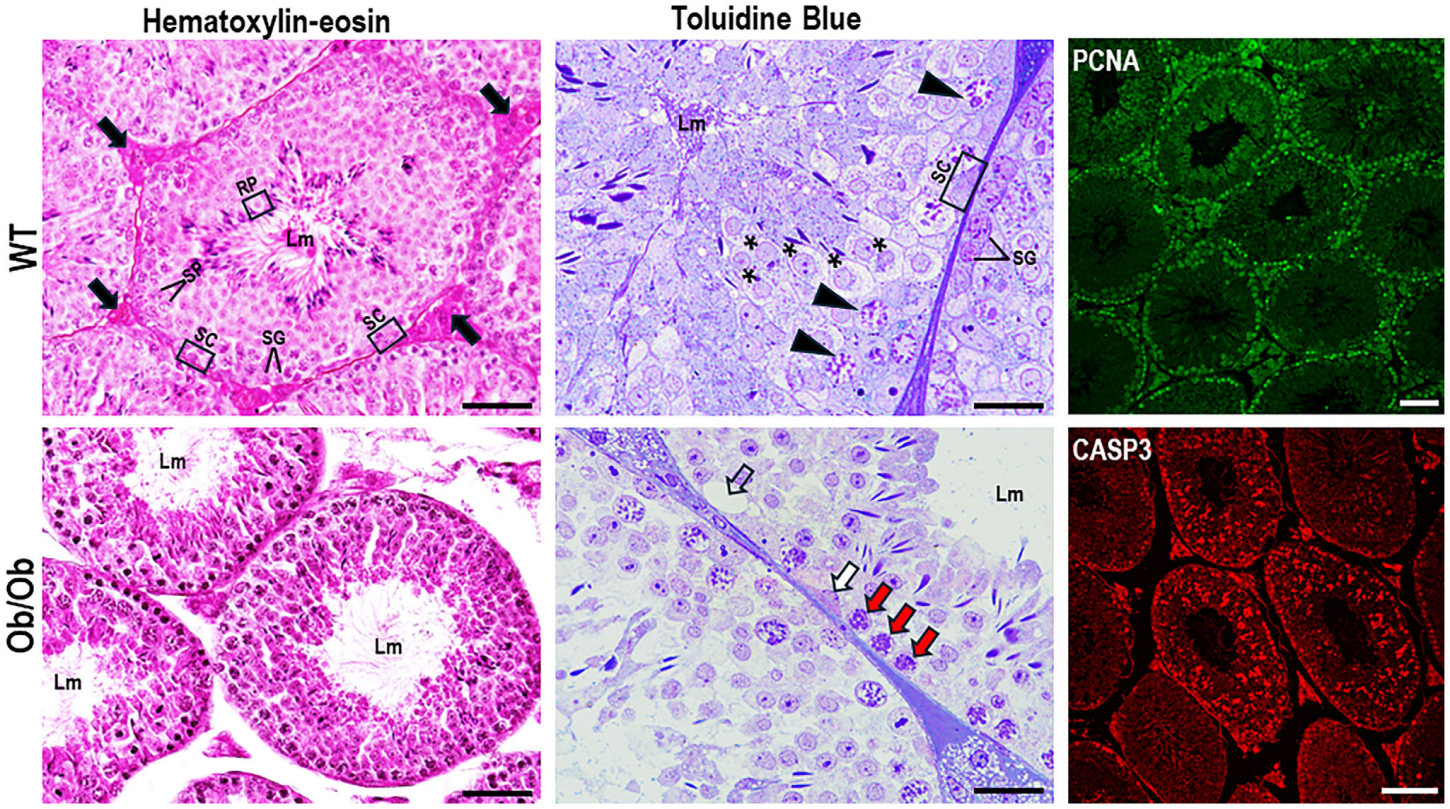
Histological sections of testis from lean mice (WT) and obese leptin knockout mice (Ob/Ob). The absence of leptin signaling, an important adipokine for male reproduction, causes significant changes in the seminiferous tubules of Ob/Ob mice. Fewer germ cells, vacuoles (open arrow), and spermatogonia with a condensed and fragmenting nucleus (red arrows) are observed in the germinal epithelium, indicating apoptosis. In addition, a reduction in the volume of Sertoli cells (white arrow) and rare sperm in the lumen (Lm) is noted. In the interstice, there are fewer Leydig cells. Cell proliferation immunofluorescence (PCNA) shows intense marking in the germinal epithelium of lean mice. On the contrary, the marker for apoptosis (Caspase 3) is strong in the seminiferous tubules of Ob/Ob mice, corroborating the histological findings and the infertility observed in these animals. arrow heads, primary spermatocytes; asterisks, secondary spermatocytes; closed arrows, Leydig cells; Lm, lumen; RP, round spermatids; SC, Sertoli cells; SG, spermatogonies; SP, spermatocytes.

**FIGURE 4:**
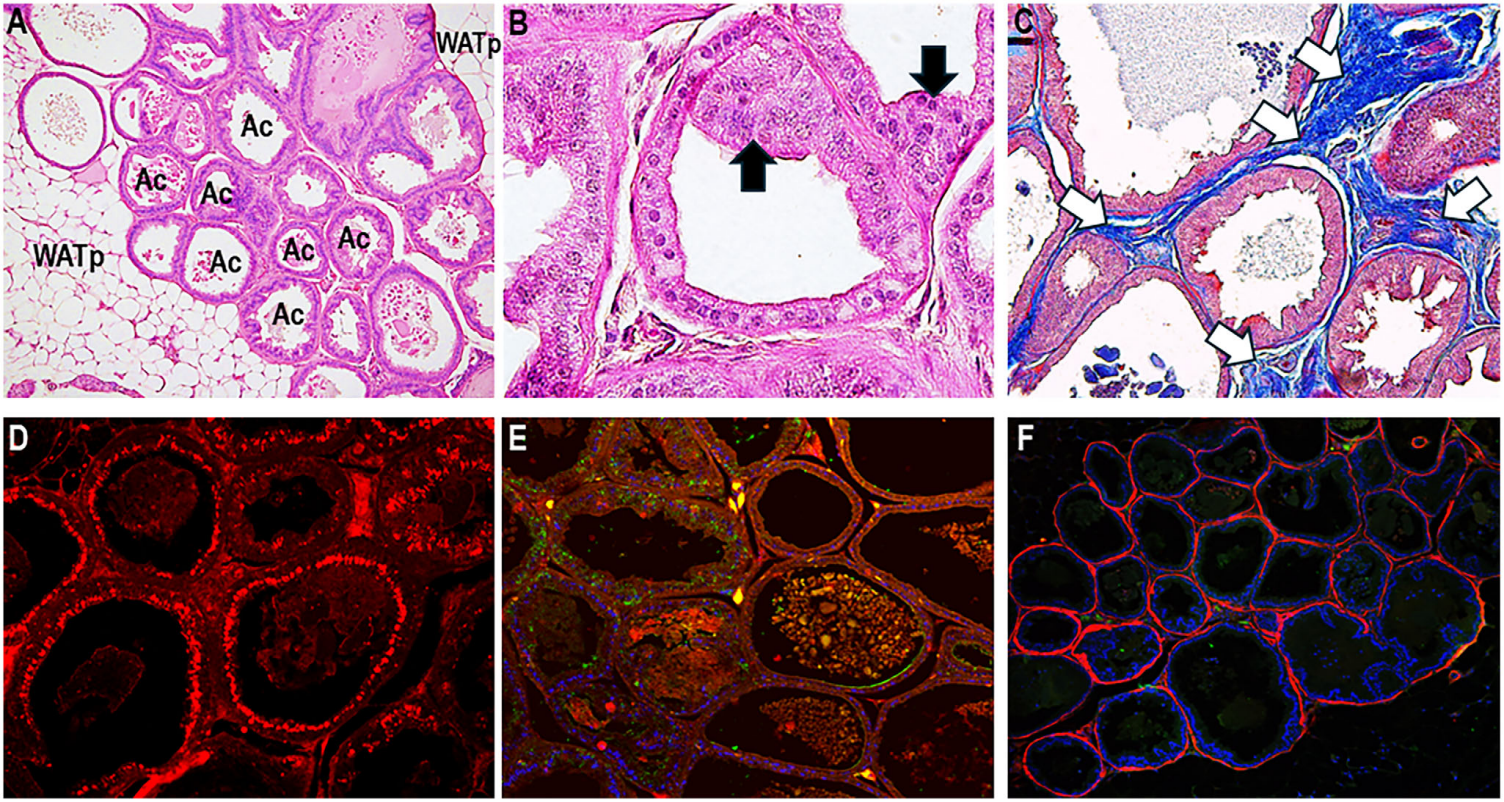
Histological sections of the prostate from obese leptin knockout mice (Ob/Ob). Note atrophied acini (Ac) with the presence of periprostatic white adipose tissue (WATp) (A), acini with intraepithelial proliferative mass indicative of prostatic intraepithelial neoplasia (PIN) (arrows), (B) increase in markers of cell death (Caspase‐3), (D), inflammation (interleukin‐ 6 and tumor necrosis factor‐alpha), (E) and adverse remodeling of the stroma with high deposition of collagen (white arrows) (C) and smooth muscle fibers (F). Hematoxylin‐eosin (A, B); Masson trichrome (C).

Leptin was discovered in 1994 through experiments that isolated it from white adipocytes, identifying it as the first adipokine of WAT. The leptin molecule has 167 amino acids, a molecular size of 16 kDa, and the tertiary structure of a globular protein [22]. Leptin signaling occurs systemically via its transmembrane receptors called OB‐Rs (OB‐Ra, OB‐Rb, OB‐Rc, OB‐Rd, OB‐Re, and OB‐Rf), which are widely distributed in different tissues, including the testis and prostate [23, 24]. The crucial role of leptin is the central coordination of metabolism, balancing energy consumption and expenditure. Its plasma levels are proportional to adiposity, therefore being an important biomarker of obesity [25]. A positive correlation exists between body mass, adiposity, leptin levels, and male reproductive failure. In a diet‐induced obesity model, increased body mass and adiposity, increased leptin levels (hyperleptinemia), and reduced plasma follicle‐stimulating hormone (FSH) and testosterone were observed [26, 27]. Leptin knockout mice (Ob/Ob) are hyperphagic, exhibit severe obesity with a high degree of adiposity, hypertrophied adipocytes, reduced testosterone levels, impairment of steroidogenic pathways, significant cellular changes in the germinal epithelium, and failures in spermatogenesis [23] (Figures 2 and 3). These changes in male reproductive health can be explained by the interface of leptin with energy metabolism and the HPG axis. The HPG axis is the center for controlling testosterone production (steroidogenesis) and maintaining male fertility. Its functioning begins in the hypothalamus, which stimulates the pituitary gland through the signaling of gonadotropin‐releasing hormone (GnRH), a hormone with a short peptide molecule that acts on the anterior lobe of the pituitary, stimulating the release of luteinizing hormone (LH) and the FSH into the bloodstream. LH and FSH are crucial for regulating testicular functions through their action on Leydig and Sertoli cells [28]. Leydig cells respond to LH signaling by converting cholesterol into testosterone (steroidogenesis), and Sertoli cells are modulated by FSH, which respond by providing support for spermatogenesis and secreting inhibin B [29, 30]. Testosterone and inhibin B return to the hypothalamus and anterior pituitary to regulate the synthesis of mediators (GnRH, LH, and FSH), thus controlling serum testosterone levels. Any change in this synchronized system of the HPG axis can cause imbalances in reproductive mechanisms and male fertility. Under physiological conditions, the HPG axis is activated by kisspeptins, peptides dependent on the action of leptin for their expression, which acts directly on the hypothalamus, regulating GnRH secretion [31]. Kisspeptin neurons are direct targets of leptin action; both kisspeptin and leptin are co‐expressed in populations of neurons in the arcuate nucleus of the hypothalamus and mediate effects on the secretion of GnRH and testosterone, being fundamental for male reproductive functions [32, 33]. In addition to central regulation of the HPG axis, leptin regulates reproductive functions by acting directly on testicular cells through passage through the blood‐testicular barrier and modulation of steroidogenesis [34]. In the testicular microenvironment, leptin binds to its receptors present on Leydig cells (OB‐Ra and OB‐Rb) and activates the canonical Janus kinase 2 (JAK2) and signal transducer and activator of transcription 3 (STAT3) pathways. The activation of JAK2 causes the phosphorylation of several residues of the OB‐Rs, which consequently activates STAT3, resulting in the translocation and transcription of steroid genes, especially those of the translocator protein (TSPO) and the steroidogenic acute regulatory protein (STAR) [33]. In the face of obesity and the expansion of WAT, leptin is hypersecreted, generating central and peripheral resistance to its action. This condition impairs the HPG axis and testicular functions. Hyperleptinemia generates a chronic state of inflammation that suppresses kisspeptin neurons and the functioning of the HPG axis, resulting in reduced levels of GnRH, LH, FSH, and testosterone [28, 35]. The obese and hyperleptinemic state increases the expression of suppressor of cytokine signaling 3 (SOCS3) at the testicular level. SOCS3 is the main inhibitor of leptin signaling; its increase inhibits the activation of the JAK2/STAT3 pathway, directly affecting Leydig cells and steroidogenesis [36, 37]. In this scenario, reproductive mechanisms fail, resulting in a decrease in the weight and volume of the testicles (hypogonadism) and a reduction in the number of Leydig cells, spermatocytes, and sperm [38]. Furthermore, androgen‐dependent organs such as the prostate are also affected by the triad of obesity, hyperleptinemia, and suppressed leptin signaling [39]. Obese mice that do not express leptin present an increase in periprostatic WAT, atrophy of the acini with epithelial changes indicative of prostatic intraepithelial neoplasia (PIN), an increase in markers of cell death (Caspase‐3) and inflammation (Interleukin‐6 and Tumor Necrosis Factor‐alpha). Additionally, they show adverse remodeling of the stroma with high deposition of collagen and smooth muscle fibers [40] (Figure 4).

## 4 Adiponectin

Adiponectin (adipocyte‐derived hormone) is a protein with a molecular weight of 30 kDa that was discovered shortly after leptin in 1995 when it was called adipocyte complement‐related protein (Acrp30) [41]. Since then, adiponectin has attracted significant attention as a messenger for connecting adipose tissue at the interface with other metabolism‐related organs [42]. In humans, adiponectin is encoded by a polypeptide of 244 amino acids and, in mice, by 247 amino acids. Adiponectin signals through two distinct receptor isoforms, AdipoR1 and AdipoR2, targeting the liver, pancreas, heart, kidneys, and tissues of the male reproductive system. Under physiological conditions, adiponectin exerts anti‐inflammatory/antifibrotic, antiapoptotic, and insulin‐sensitizing functions [43]. Studies suggest that adiponectin regulates male reproductive functions by acting on the HPG axis, passing through the blood‐brain barrier, and signaling through its receptors distributed centrally in the hypothalamus, pituitary, and peripherally in the testis [44, 45]. In the testis, the action of adiponectin and the expression of its receptors, AdipoR1 and AdipoR2, gradually increase from the prenatal period, reaching a peak during puberty, which is maintained until senescence, suggesting that the upregulation of adiponectin and its receptors is essential for testicular sexual maturation [46]. AdipoR2 is present in Leydig and Sertoli cells, as well as sperm, reinforcing the participation of adiponectin in steroidogenesis and spermatogenesis [47]. The intratesticular action of adiponectin occurs through binding to its receptors, proceeded by the activation of AMP‐activated protein kinase (AMPK) via AdipoR1 and by the activation of mitogen‐activated protein kinase (MAPK) and peroxisome proliferator‐activated receptor‐α (PPAR‐α) via AdipoR2. The activation of these pathways counter‐regulates testosterone synthesis increases spermatogenesis, and enhances sperm maturation, contributing to a healthy reproductive profile [48]. Plasma adiponectin levels are inversely correlated with WAT mass; obese men have reduced levels of adiponectin and testosterone and fertility failure. Adiponectin deficiency is reported to result in a reduction in the action of GnRH and, consequently, a disruption in the release and signaling of LH and FSH [10]. AdipoR2 knockout mice exhibit reduced testicular weight, seminiferous tubule atrophy, and aspermia [49]. Recently, it was shown that diet‐induced obese mice and AdipoR1 knockout mice present an increase in the expression of apoptotic genes and proteins in the testis, a reduction in testicular weight, and a decrease in sperm count with a reduction in sperm motility and fertilization capacity. These changes were associated with decreased AMPK signaling and increased activation of Caspase‐6 activation [50].

## 5 Resistin

Resistin was first described in 2001 by Steppan et al. [51] as a small circulating mouse protein that was specifically expressed and secreted by adipocytes (called adipose tissue‐specific secretory factor, ADSF) (Steppan et al. [52]; Steppan and Lazar [53]; Kim et al. [54]). Serum resistin levels were notably increased in mouse models of genetic and diet‐induced obesity. Later, resistin was proposed as a potential link between obesity and diabetes, with a close relationship to the development of insulin resistance (Steppan et al., [51]). Resistin is produced during adipocyte differentiation and antagonizes insulin's effects, decreasing glucose's internalization in adipocytes, muscle cells, and other tissues. Currently, resistin is known as a pro‐inflammatory adipokine that exerts its actions by binding to its receptors, including toll‐like receptor 4 (TLR4) and adenylyl cyclase‐associated protein 1 (CAP1) [55]. Resistin levels are proportional to the expansion of white adipocytes, and in obese individuals, this change in resistin status generates negative repercussions on male reproductive health. In Leydig cells, resistin activates nuclear factor kappaB (NFκB) transcription factors, negatively modulating the AMPK pathway, which leads to the suppression of c‐Jun amino terminal kinases (cJUN) and the orphan nuclear receptor (Nur77) and to activation of AP‐1 transcription factor subunit (cFos) and Dax1 (dosage‐sensitive sex reversal, adrenal hypoplasia critical region, on chromosome X, gene 1), resulting in decreased expression of the STAR and consequently, of steroidogenesis [56]. Recent in vitro studies show that Sertoli cells exposed to high concentrations of resistin (compatible with the levels found in obesity) undergo maturation interruption, remaining in the prepubertal quiescent state, which can actively affect the initiation and maintenance of spermatogenesis, resulting in fertility problems in adult life [57]. Therefore, in obesity, high levels of resistin are related to inflammation, changes in steroidogenic molecular pathways, reduced testosterone levels, and failures in spermatogenesis.

## 6 Visfatin

Visfatin, also known as nicotinamide phosphoribosyltransferase (NAMPT), is a new adipokine with effects analogous to insulin. It binds to insulin receptors and improves glucose sensitivity and tolerance. Visfatin is secreted preferentially by visceral adipose tissue; structurally, it is a protein made up of 491 amino acids and has a molecular weight of 52 kDa [58]. Recent evidence shows that visfatin is expressed in the testis under normal physiological conditions, especially in Leydig cells, spermatocytes, and sperm [59]. Similarly, visfatin was found to participate in spermiogenesis; its expression was detected in round spermatids [60]. In an in vitro model, visfatin positively stimulated Leydig cells, increasing the expression of steroidogenic enzymes and testosterone synthesis [61]. Current findings show that visfatin inhibition resulted in a reduction in the expression of androgen receptors (ARs), an increase in the secretion of estrogen and its receptors (ERs), and an increase in the expression of aromatase and apoptotic markers in the germinative epithelium, including caspase‐3 and BCL‐2 [62]. Visfatin concentrations correlate positively with body mass, testicular weight, and serum testosterone levels and negatively with plasma glucose concentrations [63]. In a model of obesity and type 2 diabetes mellitus (DM2), increased levels of visfatin were found, which were negatively correlated with several semen quality parameters and with the hormonal levels of LH and testosterone. In parallel, Kiss‐1 hypothalamic neuron mRNA was reduced, suggesting that elevated visfatin levels negatively regulate GnRH and LH secretion via down‐regulation of the Kiss‐1 system [64].

## 7 Apelin

Apelin was recognized as an adipokine in 2005; structurally, it has different isoforms ranging from 12 to 36 amino acids, all encoded by the proapelin gene (APLN). Apelin is an endogenous ligand of the specific APJ receptor, which belongs to the family of G protein‐coupled receptors [65]. Several studies report the expression of apelin and APJ in different compartments in the testis of rats, mice, and dogs, indicating that apelin plays a role in steroidogenesis and spermatogenesis [66, 67]. In humans, the expression of apelin and APJ was verified in Leydig cells, the acrosome, and the sperm's tail [68]. It has been reported that apelin secretion by adipose tissue is modulated by nutritional status, such as hunger and satiety, and its release increases via insulin stimulation, which suggests a direct association with diabetes [69]. In a gestational diabetes model, serum apelin levels were increased and predisposed the offspring to obesity with consequent functional damage to the testis due to the reduction in the expression of APJ receptors, signaling a possible testicular resistance to apelin. The persistence of this condition led the offspring to subfertility and infertility, marked by a reduction in testosterone synthesis and failures in spermatogenesis [70]. Similarly, it was shown that intracerebroventricular infusion of apelin in male rats reduced serum testosterone levels. At the same time, histological analyses showed a reduction in the number of Leydig cells, indicating that apelin may play a role in central regulation and decreased steroidogenesis via suppression of LH secretion [71].

## 8 Chemerin

Chemerin, discovered in 2003, is considered a new multifaceted adipokine. It plays crucial roles in adiposity, immunity, and energy metabolism by binding to different receptors: chemokine‐like receptor 1 (CMKLR1), G‐protein‐coupled receptor 1 (GPR1), and C‐C motif chemokine receptor‐like 2 (CCRL2) [72]. A positive correlation exists between serum chemerin and obesity‐related factors, including insulin resistance, BMI, and dyslipidemias. Chemerin knockout mice show greater hepatic gluconeogenesis and insulin sensitivity with increased glucose uptake by skeletal muscle [73]. Chemerin exerts central effects through its expression in the hypothalamus and pituitary gland, accomplishing a potential role in the control of reproductive neuroendocrine functions. Furthermore, recent studies suggest that chemerin and its receptors are expressed in the testis and play a role in reproductive mechanisms under both physiological and pathological conditions [74]. Chemerin and the receptors CMKLR1 and GPR1 are located in Leydig cells in the testis of rats and humans. In an in vitro model, chemerin suppressed the production of testosterone by Leydig cells by inhibiting the expression of 3β‐hydroxysteroid dehydrogenase (3β‐HSD) and phosphorylation of mitogen‐activated protein kinase/extracellular signal‐regulated kinase 1/2 (MAPK ERK1/2) [75]. Knockout mice for the CMKLR1 receptor showed lower plasma testosterone levels when compared to wild‐type mice. Additionally, Leydig cells from knockout animals showed reduced gene expression of steroidogenic enzymes (3β‐HSD, STAR, and p450SCC), demonstrating that chemerin and CMKLR1 exert important roles in testicular steroidogenesis [76]. Current evidence shows that seminal plasma concentration of chemerin is negatively correlated with sperm concentration and motility [77]. These findings provide new pathways to investigate the role of chemerin in spermatogenesis.

## 9 Omentin‐1

Omentin‐1, also called intelectin‐1, is a new adipokine composed of 313 amino acids with a molecular weight of 120 kDa. Its expression occurs mainly in visceral adipose tissue (omental and epicardial) but is also expressed in mesothelial cells, vascular cells, the small intestine, and the colon [78]. Omentin‐1 expression levels in preadipocytes are downregulated by glucose/insulin and stimulated by fibroblast growth factor‐21 [79]. Omentin‐1 is fundamental in maintaining body metabolism and insulin sensitivity, and it has essential anti‐inflammatory, antiatherosclerotic, cardioprotective, and antioxidant effects. Clinical studies point to circulating omentin‐1 as a biomarker of obesity and metabolic syndrome [80]. Despite its protective and beneficial effects on the morphophysiology of various tissues and systems, to date, few studies have shown the effects of this adipokine on male reproduction. Recent data showed that omentin‐1 was localized in human sperm and the tissues of the male reproductive system. It was found in semen that omentin‐1 originates from the seminal vesicles and that its levels increase in inflammatory conditions and are negatively correlated with sperm parameters [81]. For contrary reasons, the role of omentin‐1 in the cellular and molecular mechanisms of the male reproductive system deserves to be explored more extensively since this adipokine has a protective role and anti‐inflammatory properties in several tissues.

## 10 Vaspin

Vaspin, also known as SERPINA12, was first described in 2005 by isolating its cDNA from the visceral adipose tissue of Otsuka Long‐Evans Tokushima fatty (OLETF) rats, a model of metabolic syndrome [82]. Vaspin is an insulin‐sensitizing adipokine and is a member of the serpin group of proteins with serine protease inhibitory activity. Vaspin comprises 395 amino acids and has a molecular weight of 45.2 kDa [83]. In addition to adipose tissue, vaspin expression is reported in several tissues, including skin, hypothalamus, stomach, liver, pancreas, and skeletal muscle, and recently it has been reported in the testis [84]. Vaspin acts by binding to the cell surface through the 78 kDa glucose‐regulated protein (GRP78), also known as the heat shock protein family A member 5 (HSPA5) [85]. The role of vaspin in male reproductive morphophysiology is still little explored; however, in the testis, vaspin signaling was verified in Leydig cells, while GRP78 was found in both compartments, in Leydig cells, and the seminiferous tubules, indicating the participation of vaspin in spermatogenesis and the regulation of Sertoli cells [64]. Current research has shown that vaspin levels were significantly higher in a model of obesity and diabetes and that there was a negative correlation with semen quality parameters and also with the hormonal levels of LH and testosterone, indicating that vaspin plays a role in male fertility at central and peripheral levels [64, 86]. Additionally, high levels of vaspin in plasma were associated with more significant DNA fragmentation in sperm [87]. These data suggest elevated vaspin levels exert inhibitory effects on endocrine regulation and testicular function.

## 11 Asprosin

**FIGURE 5:**
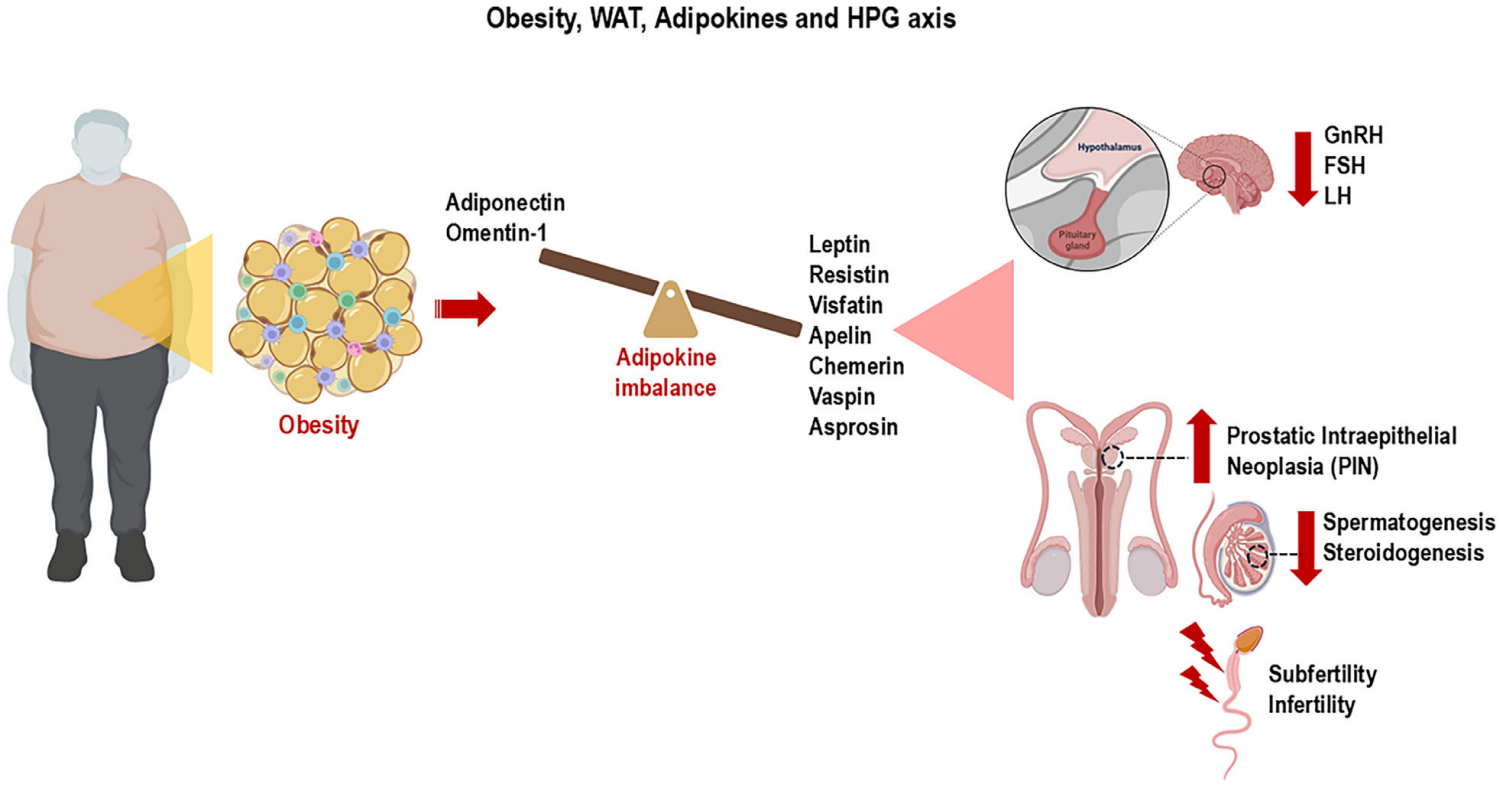
Role of adipokines in the hypothalamic‐pituitary‐gonadal axis (HPG) during obesity. Obesity induces structural and functional changes in WAT, with a consequent imbalance in the production and secretion of adipokines. While the adipokines adiponectin and omentin‐1 are downregulated, the adipokines leptin, resistin, vifastin, apelin, and chemerin are upregulated. High levels of leptin, resistin, vifastin, apelin, chemerin, vaspin, and asprosin compromise the functioning of the HPG axis, reducing the release of the hormones GnRH, FSH, and LH at the central level, generating prostate changes, failures in steroidogenesis and spermatogenesis, conditions that converge to subfertility and male infertility (Figure created with website https://app.biorender.com). FSH, hormone follicle stimulating; GnRH, gonadotropin‐releasing hormone; LH, luteinizing hormone.

Asprosin is a recent adipokine discovered and described in 2016 as a small protein with 140 amino acids and a molecular weight of 30 kDa [88]. Asprosin, a glucogenic peptide, is predominantly found in WAT, particularly in the subcutaneous compartment; however, it is also expressed in the liver, pancreas, stomach, lungs, heart, brain, and testis [88, 89]. Asprosin is a fasting‐induced hormone that promotes glucose production in the liver and stimulates appetite in the hypothalamus by activating the cAMP signaling pathway via an unknown G protein‐coupled receptor (GPCR) [90]. Furthermore, asprosin signals in different tissues by binding to the Olfr734 receptor (olfactory receptor) [90]. Asprosin controls the orexigenic response by increasing signaling from AgRP neurons and inhibiting anorexigenic POMC neurons, stimulating food intake [91]. Current findings show that asprosin plays a crucial role in obesity; its plasma levels are elevated in obese humans and mice and are correlated with insulin resistance, type 1, and type 2 diabetes mellitus [92, 93]. Recent data showed that intratesticular administration of aprosin in mice (0.1 and 1.0 µg per testis) generated positive immunoreactivity in Leydig and Sertoli cells. Furthermore, asprosin generated an increase in glucose and lactate levels, an increase in the expression of the Olfr734 receptor, the insulin receptor (IR), the glucose transporter 8 (GLUT 8), and the activity of lactate dehydrogenase (LDH). Additionally, asprosin administration increased testicular expression of cell proliferation (PCNA) and cell survival (Bcl2) and decreased germ cell apoptosis (Caspase 3), leading to increased sperm count. Treatment with asprosin also resulted in an increase in testosterone and steroidogenic markers (steroidogenic acute regulatory protein: StAR; 3beta‐hydroxysteroid dehydrogenases: 3β HSD and 17beta‐hydroxysteroid dehydrogenases: 17β HSD). Asprosin treatment promoted testicular glucose uptake and lactate synthesis to provide energy for steroidogenesis and spermatogenesis [94]. The asprosin‐receptor Olfr734 signaling axis was protective against the deterioration of sperm motility induced by a high‐fat diet [95]. According to these findings, asprosin emerges as an autocrine/paracrine regulator of testicular functions. However, studies on its mechanisms of action on male reproduction remain scarce, especially in the face of obesity and metabolic diseases. Figure 5 summarizes adipokine signaling altered by obesity in the male reproductive system.

## 12 Clinical Implications and Strategies for Positive Modulation of Adipokines

The global incidence of obesity is an emerging public health issue and directly impacts male reproductive health. The metabolic outcomes of obesity, which include changes in the adipokine secretion profile detailed above, have been directly associated with infertility in obese men [96, 97]. As a result of the imbalance of these adipokines, a vicious cycle arises: dysregulation of the hypothalamic‐pituitary‐testicular axis leads to a decrease GnRH release by the hypothalamus, resulting in a subsequen reduction in LH and FSH levels by the pituitary gland. This affects Leydig cells, attenuating steroidogenesis and thus reducing testosterone levels, further increasing adiposity due to increased lipogenesis [97]. In turn, increased adiposity increases the conversion rates of testosterone into estradiol, which causes negative feedback in the hypothalamus's arcuate nucleus, decreasing the release of kisspeptin [98]. The decrease in kisspeptin inhibits the release of GnRH, once again resulting in a decrease in the release of FSH and LH by the pituitary gland and a reduction in testosterone levels [99]. As a consequence of this vicious cycle, spermatogenesis failures and hypogonadism arise, as well as changes in prostate morphophysiology [33, 100]. This set of endocrine and structural disruptions converges in negative impacts on semen parameters, including abnormal sperm motility, viability, and morphology; changes in sperm DNA; and reduction in sperm concentration with subsequent infertility [98, 101].

Given this scenario, strategies to overcome obesity and positively modulate adipokines are necessary. In this sense, bioactive compounds (BCs) deserve attention; numerous recent studies associate the beneficial effects of BCs on WAT and the secretion of adipokines, especially phenolic compounds [102, 103, 104]. Phenolic compounds are secondary plant metabolites formed by at least one aromatic ring with one or more hydroxyl groups attached. They can be found in different food sources, such as vegetables, fruits, seeds, tea, nuts, and red wine [105].

Anthocyanins are phenolic compounds classified into six types: delphinidins, cyanidins, petunidins, pelargonidins, peonidins, and malvidins. They are found in red raspberries, blueberries, strawberries, cherries, plums, and black soybeans [106]. Anthocyanins have anti‐inflammatory, antioxidant, antidiabetic, and antiobesogenic properties. In an obesity model, anthocyanins were able to reduce body mass and adipose tissue mass, positively impacting the secretion of the adipokines leptin and resistin, corroborating the improvement of inflammation by modulating the TLR4/AMPK pathways [107]. Anthocyanins also restored the secretion of adiponectin, vaspin, and visfatin by adipose tissue, exhibiting a protective effect in the treatment of metabolic diseases [108, 109, 110]. In human adipocytes, anthocyanins increase chemerin secretion, improving insulin sensitivity and protecting against inflammation [111].

Resveratrol is a natural phenolic compound found in various foods, such as red wine, blueberries, grapes, and peanuts. Resveratrol has several pharmacological properties, including antioxidant, anti‐inflammatory, and immunomodulatory actions, as well as efficacy in the prevention and treatment of cardiovascular diseases, cancer, and obesity [112]. Supplementation with resveratrol mitigated changes in the adipose tissue of obese mice via improvement in endoplasmic reticulum stress, a reduction in the size of adipocytes, and normalization in the secretion of leptin and adiponectin, which attenuated the expression of inflammatory markers [113]. The beneficial effects of resveratrol on the secretion of adipokines were also proven in an in vitro model. 3T3‐L1 adipocytes treated with resveratrol and its metabolites (trans‐resveratrol‐3‐O‐glucuronide and trans‐resveratrol‐4'‐O‐glucuronide) showed satisfactory expressions of leptin, visfatin, and apelin [114]. In an experimentally induced diabetes model, resveratrol increased insulin sensitivity via WAT's normalization of visfatin and vaspin secretion [115].

Quercetin is a phenolic compound from the flavonoid class present in plant foods, including drinks such as tea, red wine, apples, onions, and cocoa. In an experimental model of metabolic syndrome, quercetin attenuated obesity and adipogenesis by downregulating PPARγ. Quercetin exerted anti‐inflammatory effects by restoring adipokine balance, reducing resistin secretion, and increasing adiponectin secretion [116]. In another study, quercetin associated with calorie restriction improved the balance of adipokines leptin/adiponectin in obese mice by reducing oxidative stress markers [117]. Quercetin also positively modulated chemerin, recovering its levels and anti‐inflammatory activity in a model of obesity and diabetes [118].

**FIGURE 6:**
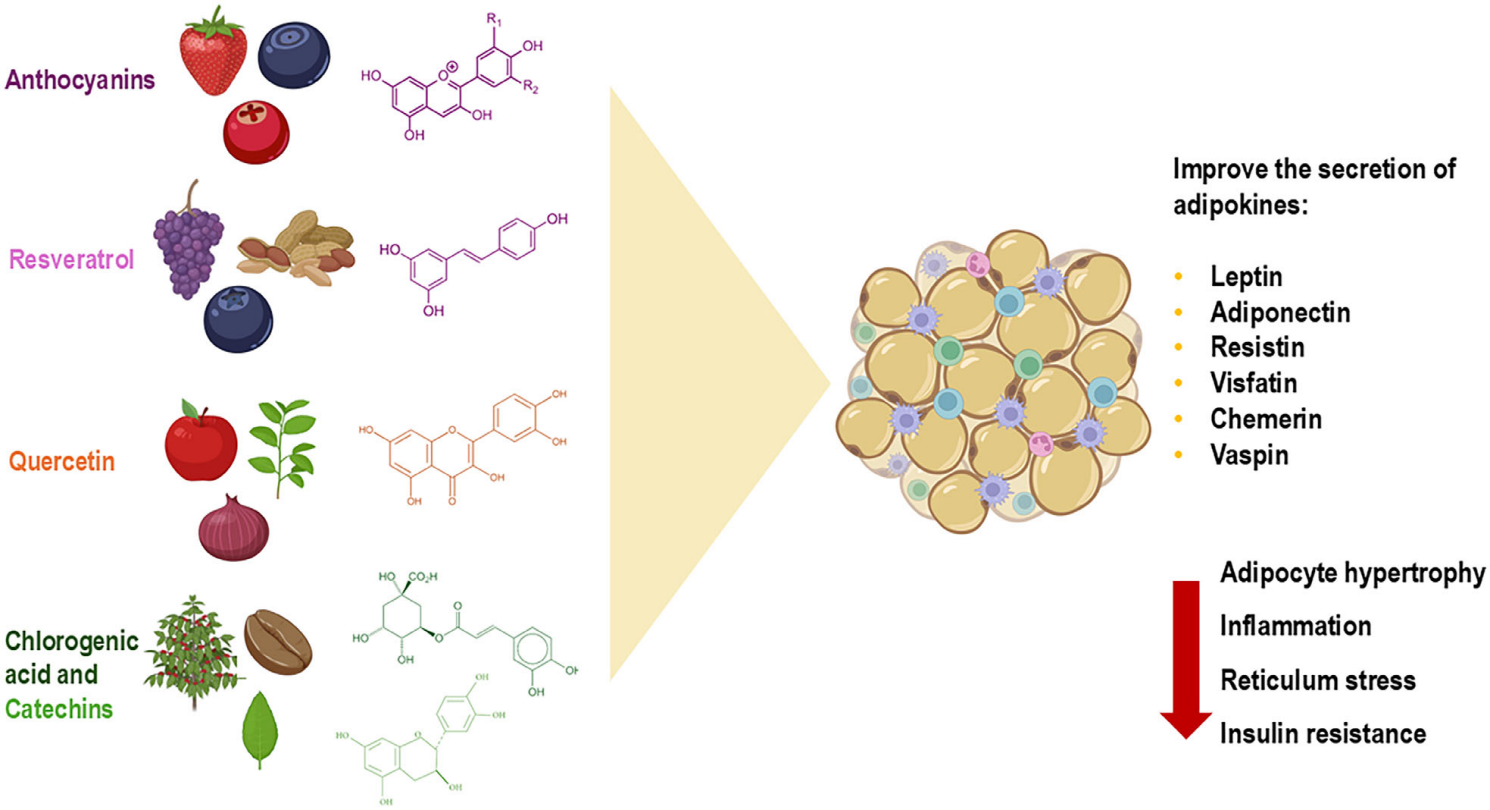
Beneficial effects of phenolic compounds on the secretion of adipokines by white adipose tissue (WAT). Anthocyanins, resveratrol, quercetin, chlorogenic acid, and catechins represent nutraceutical strategies for improving the secretion of leptin, adiponectin, resistin, visfatin, chemerin, and vaspin. As a result, there is a reduction in adipocyte hypertrophy, inflammation, endoplasmic reticulum stress, and insulin resistance (Figure created with website https://app.biorender.com).

Chlorogenic acid and catechins are phenolic compounds present in coffee and green tea, respectively. Chlorogenic acid demonstrated a potential anti‐obesity effect in high‐fat diet‐induced mice, where positive modulation of leptin and adiponectin was observed via PPAR‐alpha expression and increased beta‐oxidation of fatty acids [119]. Recently, chlorogenic acid was shown to reduce body mass and leptin resistance in overweight men [120]. Catechins reduced body mass, adiposity, and inflammation in an induced obesity model via increased adiponectin expression and reduced TLR4/TNF‐α signaling [121]. In a model of metabolic syndrome, catechins decreased food intake and body mass and normalized leptin secretion via activation of the AMPK pathway [122]. According to the above, phenolic compounds exhibit properties that positively modulate the secretion of adipokines and the loss of body mass (Figure 6), therefore being potential therapeutic targets that deserve to be investigated in the clinical management of obesity and male infertility.

## 13 Conclusion

Since the discovery of leptin, WAT has stood out for its endocrine role and interface in the systemic regulation of various organs and tissues. Obesity and changes in the adipokine secretion profile negatively impact the morphophysiology of the male reproductive system. In the current global obesity pandemic with the high rate of reproductive dysfunction in obese men, adipokines emerge as promising therapeutic targets for understanding the mechanisms that lead to subfertility and male infertility within the change in nutritional status. In this review, we show, in an updated way, the link between obesity and changes in the signaling pathways of the adipokines leptin, adiponectin, resistin, visfatin, apelin, chemerin, omentin‐1, vaspin, and asprosin in male reproduction. In conclusion, understanding the relationship between obesity, these adipokines, and reproductive dysfunction may contribute to new strategies for the treatment of male infertility.

## Conflicts of Interest

The authors declare no conflicts of interest.

## Data Availability

The data that support the findings of this study are available from the corresponding author upon reasonable request.
